# Respiratory Syncytial Virus Matrix Protein Induces Lung Epithelial Cell Cycle Arrest through a p53 Dependent Pathway

**DOI:** 10.1371/journal.pone.0038052

**Published:** 2012-05-25

**Authors:** Tao Bian, John D. Gibbs, Claes Örvell, Farhad Imani

**Affiliations:** 1 Laboratory of Respiratory Biology, National Institute of Environmental Human Science, Durham, North Carolina, United States of America; 2 Global Vaccines, Inc., Durham, North Carolina, United States of America; 3 Huddinge University Hospital, Department of Clinical Virology, Karolinska Institute, Stockholm, Sweden; 4 ViraSource Laboratories, Durham, North Carolina, United States of America; University of Iowa, United States of America

## Abstract

Respiratory syncytial virus (RSV) is the major cause of viral respiratory infections in children. Our previous study showed that the RSV infection induced lung epithelial cell cycle arrest, which enhanced virus replication. To address the mechanism of RSV-induced cell cycle arrest, we examined the contribution of RSV-matrix (RSV-M) protein. In this report, we show that in both the A549 cell line and primary human bronchial epithelial (PHBE) cells, transfection with RSV-M protein caused the cells to proliferate at a slower rate than in control cells. The cell cycle analysis showed that RSV-M protein induced G1 phase arrest in A549 cells, and G1 and G2/M phase arrest in PHBE cells. Interestingly, RSV-M expression induced p53 and p21 accumulation and decreased phosphorylation of retinoblastoma protein (Rb). Further, induction of cell cycle arrest by RSV-M was not observed in a p53-deficient epithelial cell line (H1299). However, cell cycle arrest was restored after transfection of p53 cDNA into H1299 cells. Taken together, these results indicate that RSV-M protein regulates lung epithelial cell cycle through a p53-dependent pathway, which enhances RSV replication.

## Introduction

Respiratory syncytial virus (RSV) is a major cause of respiratory tract infection in infants and young children worldwide [1], [2]. In the US, approximately 125,000 children are hospitalized annually with a 2% of mortality rate [3]. However, there are no effective vaccines for RSV. Importantly, RSV infection in early life has been associated with subsequent development and exacerbations of asthma [4], [5].

RSV, a member of paramyxoviridae family, is an enveloped virus with a single-stranded negative sense RNA genome, which replicates in the cytoplasm of host cells. The RSV genome encodes nine structural proteins and two non-structural proteins, comprising the envelope glycoproteins (F, G, and SH), the nucleocapsid proteins (N, P, and L), the nucleocapsid-associated proteins (M2-1 and M2-2), and the matrix protein (M) [6].

In our previous study we showed that RSV infection induced epithelial cell cycle arrest [7]. In addition to participating as an integral part the virus particle, RSV-M protein is shuttled to the host cell nucleus at an early stage of virus replication, where it can inhibit cellular transcription [8], [9], [10], [11]. Therefore, we hypothesized that RSV-M protein played a key role in the RSV-induced cell cycle inhibition and therefore enhanced virus replication.

To further delineate the mechanism of RSV-M protein induction of cell cycle arrest, we examined the contribution of the tumor suppressor protein p53, which plays a pivotal role in cell cycle arrest [12]. Once activated, p53 binds cellular DNA and induces expression of several genes including GADD45, IGF-BP3 and WAF1/CIP1 which encodes p21. p21 is a key molecule in cell cycle regulation binds to and inhibits the activity of cyclin-dependent kinase (CDK) complexes, thereby inhibiting Rb phosphorylation. The phosphorylation of Rb is a well-described regulator of the cell cycle. Therefore, over expression of p53 causes arrest of cell growth [13].

Virus infections are known to activate the p53 pathway. Infection of influenza A virus, Epstein–Barr virus (EBV), adenovirus, HIV-1 and minute virus induce host cell accumulation of p53, cell cycle arrest and apoptosis [14], [15], [16], [17], [18], [19], [20]. Over-expression of p53 also is observed in chronic hepatitis C virus infected patients, suggesting an impaired cell cycle progression that lead to worsening of the disease [21]. However, some viral proteins, particularly in DNA viruses, form complexes with p53 and reduce effective p53 levels [22]. Our previous study showed that RSV infection induced lung epithelial cell cycle arrest which subsequently enhanced RSV replication [7].

Furthermore, viral proteins such as non-structural protein of influenza A virus, large T antigen of the human polyoma virus, JC virus large T protein, human parvovirus B19 NS1 protein and avian reovirus p17 protein induce cell cycle arrest in G1 or G2 phase through p53 or p21 accumulation [23], [24], [25], [26], [27]. Interestingly, the fusion protein of RSV (RSV-F) triggers p53-dependent apoptosis [25]. In our current study, our data showed that RSV-M protein induced lung epithelial cell cycle arrest through the accumulation of p53 protein.

## Material and Methods

### Plasmid Construction and Transfection

The eukaryotic expression vector pSG-5 was purchased from Stratagene (Santa Clara, CA). The RSV-M protein gene open reading frame (ORF) was amplified from RNA isolated from RSV-A2-infected cells by RT-PCR with the forward primer: 5′-TGC*GGATCC*ATGGAAACATACGTGAAC-3′; the reverse primer: 5′-TGC*GGATCC*TTAATCTTCCATGGGTTTG-3′. After digestion with restriction enzyme BamHI, the RSV-M gene was cloned into pSG-5 (pSG-M) using standard techniques. Sequencing was performed to verify the fidelity on the cloned cDNA. pcDNA3.1-p53 plasmid expressing the wild type human p53 was kindly provided by Dr. Michael Resnick (NIEHS, NIH). For transfections, DOTAP liposomal transfection reagent (Roche, Indianapolis, IN) was used for transfection DNA into A549 cells. Nucleofector kit (Lonza, Walkersville, MD) was used for transfection into PHBE cells with program W-001 according to the manufacturer’s instructions. The plasmid expressing GFP (pmaxGFP) (Lonza, Walkersville, MD) was used to evaluate transfection efficacy.

### Cells and Cell Culture Reagents

Primary human bronchial epithelial (PHBE) cells and serum-free bronchial epithelial basal medium with growth supplements were purchased from Lonza (Walkersville, MD). The human alveolar epithelial cell line A549 and human p53-deficient lung cancer cell line H1299 were purchased from American Type Culture Collection (ATCC Number: CCL-185 & CRL-5803), which were grown as a monolayer in Dulbecco’s modified Eagle’s/Ham’s F-12 medium with 5% fetal calf serum and 1% penicillin-streptomycin at 37°C in a 5% CO2 humidified incubator. H1299 is a human lung lymph node-derived non-small cell carcinoma cell line which has a homozygous partial deletion of the TP53 gene and does not express p53 protein. The p53 expressing H1299 (p53^+/+^) cell line was derived from H1299 after transfection with pcDNA3.1-p53 plasmid. G418 (Sigma-Aldrich, St. Louis, MO) selection was used to isolate stably expressing cells with the wild type p53 protein. Nutlin-3, a cell cycle regulatory compound through accumulation of p53, was purchased from Sigma-Aldrich (St. Louis, MO), diluted in captisol (CyDex, Kansas City, MO), and was used at of 10 µM.

### Virus and Infections

The human RSV subtype A2 was grown and titrated by standard plaque assay in human epithelial cell line HEp-2 as previously described [28]. After cells reached 70% confluency, they were infected with RSV at the indicated multiplicity of infection (MOI). Cells were then harvested at various times and were used for analysis.

### Cell Proliferation Assay and Cell Cycle Analysis

For cell proliferation assays, A549 and PHBE cells were transfected with pSG-M, and pSG-5 as vector control. Cells were treated with nutlin-3 as positive control for p53-induced cell cycle arrest. Cells were treated captisol alone as the vehicle control. At indicated time-point post-treatment, cells were washed and resuspended in phosphate-buffered saline (PBS). Total cell numbers at each day were determined with a hemocytometer, and dead cells were identified by trypan blue staining. Cell cycle analysis was performed by flow cytometry. Briefly, 4 hr prior to harvesting cells were treated with 10 µM bromodeoxyuridine (BrdU) (BD Bioscience, San Jose, CA). Cells were then fixed with 70% ethanol for 30 min at −20°C and then labeled with fluorescein isothiocyanate-conjugated anti-BrdU antibody according to the manufacturer’s instructions (BD Biosciences, San Jose, CA). The cell cycle properties were then analyzed using BrdU incorporation and 7-Amino-actinomycin D (7-AAD) (BD Biosciences, San Jose, CA) staining. Flow cytometry was carried out on a Becton-Dickinson FACSort flow cytometer, and quantification of cell cycle distribution was determined using either CellQuest or Modfit software (BD Biosciences, San Jose, CA).

### Western Blot and Antibodies

For western blot analysis, cells were washed twice with PBS and were then lysed in Laemmli sample buffer containing 2.5% β-mercaptoethanol. The total cellular proteins were denatured and reduced by heating at 95°C for 5 min. Protein concentration was then determined by the Bio-Rad protein assay (Bio-Rad, Hercules, CA). Proteins were resolved by SDS-polyacrylamide gel electrophoresis and were electrotransferred onto nitrocellulose membranes. The membranes were probed with respective primary antibodies and horseradish peroxidase-conjugated secondary antibody according to the manufacturer’s instructions. Primary antibodies were anti-p53 (DO-1), anti-Phospho-p53 (serine-15), anti-p21, anti-phospho-Rb (p780) and anti-glyceraldehyde-3-phosphate dehydrogenase (anti-GAPDH) (Cell Signaling, Danvers, MA); anti-RSV (Fitzgerald Industries, Concord, MA). Monoclonal antibody C781 is specific for M protein [9]. The enhanced chemiluminescence (ECL) western blot detection system was then used to visualize the immunoblotted proteins (GE Healthcare, Piscataway, NJ). Quantification of the bands were performed using ImageJ software.

### Quantitative RT-PCR

Total RNA was isolated with TRIzol RNA isolation reagent (Invitrogen, Carlsbad, CA) following the manufacturer’s instructions. RNA was reverse transcribed into first-strand cDNA using Superscript reverse transcriptase (Invitrogen, Carlsbad, CA) with oligo (A) primer. The cDNA was then amplified by real-time PCR (RT-PCR) as previously described [7]. All RT-PCR results were normalized according to GAPDH before analysis. Primer sequences used in RT-PCR were as follows: GAPDH (forward) 5′-GGACCTGACCTGCCGTCTAG-3′ (reverse) 5′-TAGCCCAGGATGCCCTTGAG-3′; RSV-NS1 (forward) 5′-AGAGATGGGCAGCAATTCAT-3′ (reverse) 5′-CACAAACACAATGCCATTCA-3′; RSV-NS2 (forward) 5′-ATCAATTCAGCCAACCCAAC-3′(reverse) 5′-ATGTGGCCTGTTTTTCATCA-3′; RSV-P (forward) 5′-GGCAAGACTCAGGAATGAGG-3′ (reverse) 5′-GTTGGATGATTGGGTTGGTT-3′; RSV-M (forward) 5′-AAAGACGATGACCCTGCATC-3′ (reverse) 5′-TGGTAAATTTGCTGGGCATT-3′; RSV-G (forward) 5′-CAACCCCAACATACCTCACC-3′ (reverse) 5′-GTGGATTGCAGGGTTGACTT-3′; RSV-N (forward) 5′-AGGATTGTTTATGAATGCCTATGGT-3′ (reverse) 5′-GCTTTTGGGTTGTTCAATATATGGTAC-3′; RSV-L (forward) 5′-CAGCCAAATCCAACCAACTT-3′ (reverse) 5′-AATTCCCTGCTCCTTCACCT-3′ and p53 (forward) 5′-TCAACAAGATGTTTTGCCAACTG-3′ (reverse) 5′-ATGTGCTGTGACTGCTTGTAGATG-3′.

### Statistical Analysis

Statistical analysis was performed on cell proliferation assays, cell cycle analysis, quantification of bands in western blots, RT-PCR and plaque assays. The values are standard errors of the means based on Student’s *t* test. Significance values (*P* values) of less than 0.05 were interpreted as statistically significant.

## Results

### RSV-M Protein Expression Inhibits Epithelial Cells Proliferation

**Figure 1:**
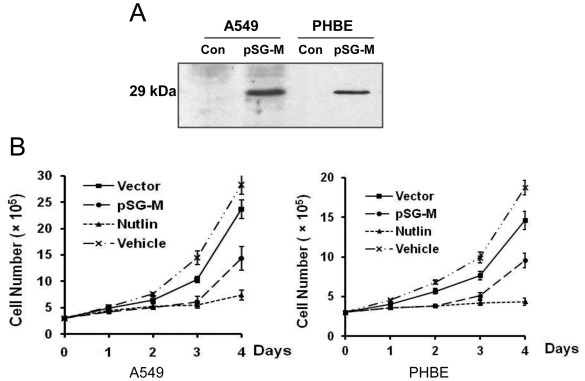
RSV-M protein expression inhibits cell proliferation. Panel A, western blot analysis, with specific anti RSV-M monoclonal antibody (C781) in cells transfected with pSG-5 plasmid alone (Con) or pSG-5 plasmid carrying RSV-M cDNA (pSG-M). Panel B, sub-confluent monolayer (approximately 10% confluency) of A549 cells (left panel) and PHBE cells (right panel) were transfected with pSG-M and pSG-5 (vector control). Cells were treated with nutlin-3 (positive control) and captisol alone (vehicle control). Cell replication was then determined by counting the cell numbers using a hemocytometer. Error bars indicate standard error of the mean (SEM) (n=3).

To assess the role of RSV-M in cell cycle regulation, we first determined the expression of RSV-M in A549 and PHBE cells by western blot analysis (Fig. 1 A). The data showed that a protein of approximately 29 KDa was expressed after transfection, which reacted with a specific mAb to RSV-M protein [10]. The transfection efficiencies are 90–100% in A549 cell and 70–80% in PHBE according to the GFP expressing plasmid (data not shown).

Next, to determine the effect of RSV-M protein expression on cell proliferation, we transfected both A549 and PHBE cells with the vector alone or with the vector carrying RSV-M. After indicated times post transfection, cells were quantified by enumeration using a hemocytometer (Fig. 1B). The results showed that pSG-M transfected cells replicated at a significantly slower rate than cells transfected with the vector alone (pSG-5) or treated with the vehicle control (captisol) (Fig. 1B). The cells treated with nutlin-3 were used as a positive control, which have similar proliferation rate as pSG-M transfected cells. The number of dead cells did not have significant difference, and were <1% in all samples.

### M Protein Expression Induces Cell Cycle Arrest

**Figure 2:**
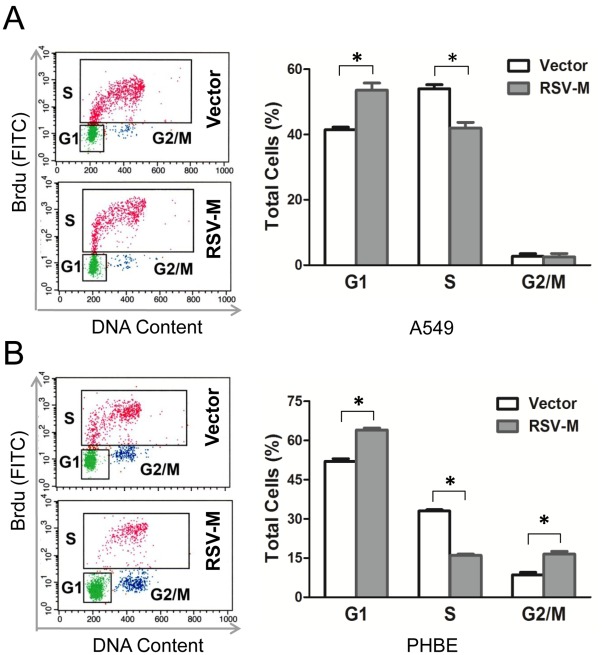
RSV-M protein induces cell cycle arrest in both A549 and PHBE cells. Cells were transfected with pSG-M or pSG-5 (vector control). The distribution of cell cycle phases of the transfected A549 cells (panel A) and PHBE cells (panel B) were determined by flow cytometry at 2 days post-transfection. The cell cycle properties were analyzed using BrdU incorporation and 7-AAD staining. The left panels are FACS data, and the right panels are the statistical analysis of each cycle from 3 separate experiments. Error bars are the standard error of the mean (n=3). *p-values<0.05.

Since there was a significant reduction in cell proliferation, we next performed cell cycle analysis using BrdU incorporation and flow cytometry. Data showed that in A549 cells pSG-M transfection induced a significant G1 phase arrest (G1 phase: 51.4±2.2% vs. 44.7±1.3%) (Fig. 2 A). However, in PHBE cells, both G1 and G2/M phase arrest were observed (G1 phase: 67.1±0.5% vs. 54.5±1.0%; G2 phase: 15.5±1.2% vs. 8.6±0.9%) (Fig. 2B). The data showed that RSV-M alone was sufficient for induction of cell cycle arrest.

### M Protein Expression Activates the p53 Pathway

**Figure 3:**
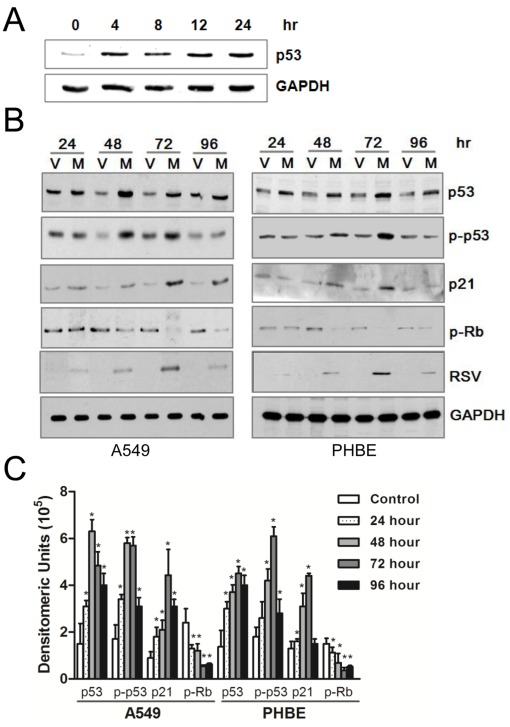
RSV Infection and RSV-M protein expression induce p53 activation. Panel A, A549 cells were infected with RSV at MOI of 5 PFU/cell. Cell extracts were prepared at indicated times and used in western blot using anti p53 mAb (DO-1). A549 cells (panel B, left) or PHBE cells (panel B, right) were transfected with pSG-M (M) or pSG-5 (V). At indicated time points, total cell extracts were prepared and were used in western blot analysis. The specific anti-total p53 (DO-1), anti-phospho-p53 at serine-15 residue, anti-p21, anti-phospho-Rb at residue 780 and anti-RSV-M protein antibody were used to identify the respective molecules. Anti-GAPDH antibody was used as an internal control. Panel C, qualification of p53, phospho-p53, p21, and phospho-Rb was performed by using ImageJ software. Error bars are the standard error of the mean (n=3). *p-values<0.05.

To **first** determine whether RSV infection induced p53 we performed a kinetic study **in** infected A549 cells. Western blot analysis showed that RSV infection induced p53 in a time dependent manner (Fig. 3A). This induction, we reasoned, maybe in part due to RSV-M protein expression. To investigate whether p53 induction and activation of downstream signaling molecules was in part due to RSV M expression, we performed transfection studies.

A549 cells and PHBE cells (Fig. 3, panel B) were transfected with pSG-M and pSG-5 (vector control). At indicated time-points, cell extracts were prepared and were subjected to western blot analysis using mAb to p53 (DO-1) and phospho-p53 (serine-15). Data showed that RSV infection increased p53 expression and phosphorylation in a time-dependent manner.

Next, we examined p53 downstream proteins p21 and phospho-Rb. Data showed that p21 accumulated significantly starting at 24 hr and reached maximal level at 72 hr post transfection (Fig. 3B and C). The level of Rb phosphorylation was diminished after RSV-M protein transfection, suggesting that downstream signals are involved in cell cycle arrest. The level of RSV-M protein expression was coincident with the maximal effect on changes in the level and the phosphorylation state of the signaling molecules. Quantification of the bands was performed using the ImageJ software, and the data showed significant time-dependent changes after RSV-M transfection as compared to vector control (Fig. 3C).

### p53 is Necessary for RSV-M Protein Induced Cell Cycle Arrest

Infection with several different viruses causes p53 activation, which is critical for cell cycle regulation. We next investigated the role of p53 in the RSV-M protein induced cell cycle arrest. To this end, a p53-deficient cell line (H1299) was used for transfection studies.

**Figure 4:**
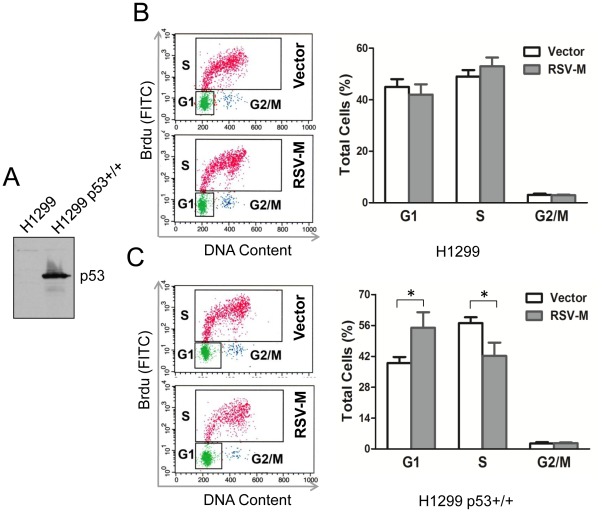
p53 is necessary for RSV-M-induced cell cycle arrest. Panel A**,** western blot analysis of H1299 cells transfected with a plasmid carrying the wild type p53 cDNA. After isolation of stable transformants, H1299 cells (panel B) and H1299 p53^+/+^ cells (panel C) were transfected with pSG-M (RSV-M) and pSG-5 (vector). The cycle phase distribution was then analysed by flow cytometry at 48 hr post transfection. The right panels are the statistical analysis of each cycle from 3 separate experiments. Error bars are the standard error of the mean (n=3). *p-values<0.05.

First, the H1299 were transfected with a plasmid carrying the wild-type (wt) p53 (pCDNA3.1-p53) or with the vector alone (pCDNA3.1). The stable H1299 p53^+/+^ and H1299 cell lines were selected with G418. The expression of p53 was then assessed by western blot analysis (Fig. 4A). Transfections studies were performed with pSG-M and the empty vector. The data from cell cycle analysis showed that in H1299 expressing of M protein alone did not induce the cell cycle arrest (G1 phase: 43.0±5.1% vs. 45.9±3.5%) (Fig. 4B). On the other hand, in H1299 p53^+/+^ cells, the pSG-M transfection induced the G1 phase arrest (G1 phase: 53.3±10.3% vs. 39.9±2.7%) (Fig. 4C). These results showed that RSV-M protein induced cell cycle arrest is dependent on p53 expression.

### Wild Type p53 Expression Enhances RSV Replication

**Figure 5:**
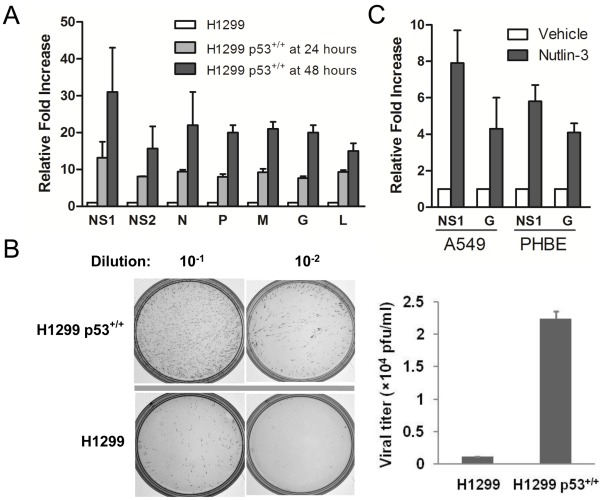
p53 expression enhances RSV replication. Panel A, H1299 and H1299 p53^+/+^ cells were infected with RSV at MOI of 1 PFU/cell. After 24 and 48 hours, total cellular RNA was isolated. The RSV gene transcription levels between the p53-expressing and non-expressing cells were normalized to GAPDH and compared at the respective time points. Panel B, cells were prepared as in panel A but to measure virus replication a standard plaque assay was performed (left panel). The number of plaques and the titer of the virus was then determined (right panel). Panel C, A549 and PHBE cells were treated with nutlin-3 or captisol alone (vehicle control). After 48 hours, cells were infected with RSV at MOI of 1 PFU/cell. After 24 hr, relative changes of NS1 and G genes were determined. The error bars are standard error of the mean from three independent assays.

Replication of several viruses is enhanced by p53 [29], [30]. Therefore, we next examined the effect of p53 on RSV replication. We tested the RSV transcription and replication in H1299 and H1299 p53^+/+^ cell lines. First, RSV transcription level was determined by RT-PCR utilizing the RSV-NS1, NS2, N, P, M, G and L gene-specific primers. RT-PCR results showed that at 24 hr post infection RSV genes mRNA levels are approximately 10 fold higher in H1299 p53^+/+^ cell line than H1299 cells (Fig. 5A). The enhancement increases to approximately 20 fold at 48 hr post infection (Fig. 5A). Next, to directly measure the effects of p53 expression on RSV replication, virus yield was determined by plaque assay. Plaque assay results showed that virus titer was approximately 20 fold higher in H1299 p53^+/+^ cell line (Fig. 5B). Further, induction of p53 in A549 and PHBE cells with nutlin-3 significantly enhanced transcription of RSV NS1 and G genes. (Fig. 5C). The results suggest that the p53 protein enhances RSV replication.

## Discussion

A number of viruses affect the cell cycle to subvert host-cell functions and increase their own replication. The cell cycle arrest during virus infection has adverse effects on lung epithelium and contributes to impede lung repair, which is associated with airways injury and bronchiolitis [31], [32]. This study for the first time demonstrates that the RSV-M protein causes the epithelial cell cycle arrest by a p53 dependent pathway.

**Figure 6:**
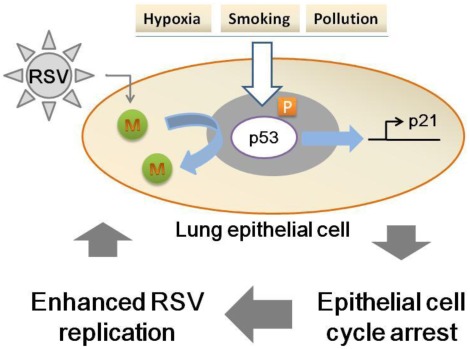
A working model of the effects of environmental factors on p53 expression and enhancement of virus replication.

Modulation of cell growth is a common feature of virus infections and can be induced by specific viral proteins [26], [27], [33]. The current study demonstrated that the epithelial cells transiently expressing RSV-M protein grew at a slower rate than cells transfected with an empty vector (Fig. 1). Effect of RSV-M protein expression was similar to the effect of nutlin-3, a known cell cycle regulator (Fig. 1). The effect of RSV-M protein on cell cycle was maximal at day three post transfection which was coincident with the peak of p53 accumulation (Fig. 3), this suggests that RSV-M protein affects cell cycle through a p53-dependent pathway. Despite the increase in p53 expression, there was no significant difference in the number of dead cells between RSV-M protein expressing cells or cells transfected with vector alone, suggesting that the changes in cell numbers is not due to cell death either by necrosis or apoptosis (data not shown).

To further determine the effect of p53 on RSV-M protein regulation of cell cycle, we used H1299 cells which are deficient in p53 expression. Transient transfection studies using H1299 cells showed that p53 was required for the RSV-M protein-induced cell cycle regulation (Fig. 4). RSV-M protein is known to be transferred to the nucleus where it can induce cellular transcriptional perturbations. Since p53 is a cell stress response factor, it is likely that transcriptional perturbation induced by RSV-M protein regulate p53 expression.

Under non-stressed conditions, p53 is a short-lived protein [34]. However, when p53 is activated by a variety of stress stimuli including DNA damage and virus infections [35], it is rescued from degradation and then translocated into the nucleus. In the nucleus, it can be phosphorylated on serine-15 by ataxia ATM (telangiectasia-mutated) or ATR (ATM and Rad3-related) proteins which causes the initiation of a cascade of subsequent post-translational modifications [36]. One important p53 targets is p21 cip1/waf1 which is a direct mediator of cell cycle arrest at the G1 and G2/M phase. In turn, p21 can complex with specific Cdks resulting in inhibition of Rb phosphorylation. The phosphorylation of Rb is a well-described regulator of cell proliferation by controlling progression through the cell cycle restriction points [37]. Alteration in Rb phosphorylation is widely used as an indicator of G1/S phase perturbations. In our experiments, results of western blots showed the expression of RSV-M protein induced accumulation of p21 and reduction in Rb phosphorylation (Fig. 3).

The cell cycle analysis on RSV-infected cells or RSV-M protein transfected cells showed differences between A549 cell line and primary human epithelial cells. In A549 cells, the cell cycle was arrested in the G1 (Fig. 2A), whereas both G1 and G2 phases were arrested in the PHBE cells (Fig. 2B). Our current data obtained in infected A549 cells are consistent with the cell cycle arrest by RSV infection that we previously reported [7]. However, in PBHE cells, in addition to G2/M arrest RSV-M transfection also induced G1 arrest. At this point we do not know the exact reason for this discrepancy but it may be due to more complex events that take place during virus infection as compared to transfection with one protein alone. Also, during transfection, soluble M protein may be present at higher levels as compared to the levels during RSV infections where it can form complexes with other viral proteins [38]. Alternatively, it may be due to differences in the nature of each PHBE cell lot, which are isolated from different donors. These differences include but are not limited to sex, age and the genetic backgrounds of each donor. Results of further studies comparing these variables are required to provide clearer answers for the discrpencies in using different lots of PHBE cells.

The effect of p53 on virus replication has been previously reported. p53 protein promotes adenoviral and CMV replication and limits poliovirus and vesicular stomatitis virus replication [29], [30], [39], [40]. In this study, we demonstrated that p53 expression significantly enhanced the RSV replication, although the exact underling mechanism is still unclear. It is possible that inhibition of the cell cycle results in increased availability of necessary components for viral assembly. It is also possible that p53 alone or in addition to other transcription factors act as an enhancing factor for viral replication through a transcriptional effect.

It appears that the nuclear shuttling of viral proteins affecting host cell biological activity is an important mechanism to enhance virus replication. Research on nuclear shuttling protein p17 of avian reovirus showed that it facilitates virus replication by initiation of p53-dependent G2/M arrest [24], [33]. In addition, EBV nuclear antigen 3C targets p53 and modulates apoptotic activities, and HIV-1 nuclear protein Vpr induces cell cycle arrest [41], [42], [43].

Chulu et al. reported that during reovirus infection, the cell cycle blockade promotes virus growth by diverting the cellular machinery required for normal cell replication [24]. During HIV-1 and CMV infections, cell cycle is arrested at G1 or G2/M phase which creates a favorable environment for viral replication [43], [44], [45]. Furthermore, Julia et al. reported that RSV fusion protein triggered p53-dependent apoptosis [25].

In conclusion, our data suggests that RSV infections and expression of the M protein profoundly affect cell cycle through a p53-dependent pathway. This suggests that any environmental factor that can increase p53 such as air pollution, smoking and ozone could enhance viral replication (Fig. 6). In addition, regulation of cell cycle during RSV infection may play a role in damage and recovery during such infections. It is important to note that RSV is the most common virus associated with severe bronchiolitis [46]. Further experiments are underway to determine the interaction between RSV-M protein and p53 regulation on RSV replication and respiratory damage.
